# Reducing injection discomfort in dermatology outpatient clinics

**DOI:** 10.1002/ski2.402

**Published:** 2024-05-30

**Authors:** Mohammad Alzaid, Faisal R. Ali

**Affiliations:** ^1^ University of Manchester Medical School Manchester UK; ^2^ Mid Cheshire NHS Foundation Trust Macclesfield UK; ^3^ Dermatological Surgery & Laser Unit St John's Institute of Dermatology Guy's Hospital Cancer Centre Guy's and St Thomas' NHS Foundation Trust London UK

Dear Sir,

We read with interest the use of vibration devices to reduce pain of intralesional corticosteroid therapy (ICT) for alopecia, particularly in needle phobic and younger patients.^1^ There are few other readily deployable strategies that could be used in outpatient clinic. Dermatologists should not underestimate the impact of free and simple distraction methods in reducing peri‐procedural anxiety and ultimately pain perception. Engaging in gentle distractive conversations (talkaesthesia), whilst confidently performing ICT can reduce anxiety, hasten the passage of time, and reduce self‐reported perception of discomfort.^2^ Alternatively, using visual or auditory distractions of patients' choosing (such as podcast/video on their own smart device) may enhance patients' experience and reduce perceived pain. A randomized trial of 144 children undergoing invasive procedures such as venepuncture and subjected to visual and/or auditory distraction techniques concluded that such techniques are effective with no difference between them in terms of reducing pain and medical fear on Wong‐Baker faces pain rating and child medical fear scales, respectively.^3^ Moreover, skin cooling with ice is inexpensive, fast‐acting (unlike topical anaesthetic), and readily available method to dull pain associated with syringe needle insertion.^4^ Careful attention to the insulin syringes utilized to deliver the ICT, with preference to smaller gauge (e.g. 30‐gauge) and fresh needles (when possible) is recommended to avoid unnecessary pain.^5^ Frequently changing the needle ensures a sharper tip that requires less force to puncture the skin and activates fewer pain fibres.^6^ Finally, dermatologists may consider regional nerve block of the scalp, especially when ICT is needed for an extensive area.^7^

## CONFLICT OF INTEREST STATEMENT

None to declare.

## AUTHOR CONTRIBUTIONS

**Mohammad Alzaid**: Formal analysis (equal); writing – original draft (lead); writing – review & editing (lead). **Faisal R. Ali**: Conceptualization (lead); data curation (equal); formal analysis (equal); investigation (equal); methodology (equal); project administration (lead); supervision (lead); validation (equal); visualization (equal); writing – original draft (equal); writing – review & editing (equal).

## FUNDING INFORMATION

This article received no specific grant from any funding agency in the public, commercial, or not‐for‐profit sectors.

## ETHICS STATEMENT

Not applicable.

## Data Availability

Data sharing is not applicable to this article as no new data were created or analyzed in this study.
